# 3, 2, 1, go! *Cryptosporidium* counts down to sex

**DOI:** 10.1371/journal.pbio.3001638

**Published:** 2022-05-12

**Authors:** Aaron R. Jex, Christopher J. Tonkin, Stuart A. Ralph

**Affiliations:** 1 Population Health and Immunity Division, The Walter and Eliza Hall Institute of Medical Research, Parkville, Victoria, Australia; 2 Faculty of Veterinary and Agricultural Sciences, The University of Melbourne, Parkville, Victoria, Australia; 3 Infectious Diseases and Immune Defence, The Walter and Eliza Hall Institute of Medical Research, Parkville, Victoria, Australia; 4 Department of Medical Biology, The University of Melbourne, Parkville, Victoria, Australia; 5 Department of Biochemistry and Pharmacology, Bio21 Molecular Science & Biotechnology Institute, The University of Melbourne, Parkville, Victoria, Australia

## Abstract

Cryptosporidium is a leading cause of death from childhood diarrhoea, but its biology is poorly understood. This Primer explores a recent study in PLOS Biology that reveals hitherto unknown aspects of the parasite’s life cycle that may lead to improvements in ex vivo culture.

## Teaser

*Cryptosporidium* is a leading cause of death from childhood diarrhea, but its biology is poorly understood. A recent study in *PLOS Biology* reveals hitherto unknown aspects of the parasite’s life cycle that may lead to improvements in ex vivo culture.

Diarrhea is a leading cause of death and disease burden in children worldwide, particularly in low- to middle-income countries. Recent studies show that species of *Cryptosporidium* are a leading global cause of diarrheal burden in children under 5 [1–3]. Human cryptosporidiosis is primarily caused by *Cryptosporidium parvum* and *Cryptosporidium hominis*, and infection occurs through ingestion of environmentally resilient oocysts. Although causes of death from diarrheal diseases are not always well delineated, the most recent estimated annual deaths due to cryptosporidiosis range from 24,600 to 81,900 [3]. The global burden of cryptosporidiosis, as measured in disability-adjusted life years (DALYs), was 4.2 million in immediate impact and 7.85 million when chronic conditions, such as stunting, were included [3]. These findings sparked renewed interest in developing treatments for cryptosporidiosis, for which there are no vaccines nor broadly efficacious drugs.

**Fig 1:**
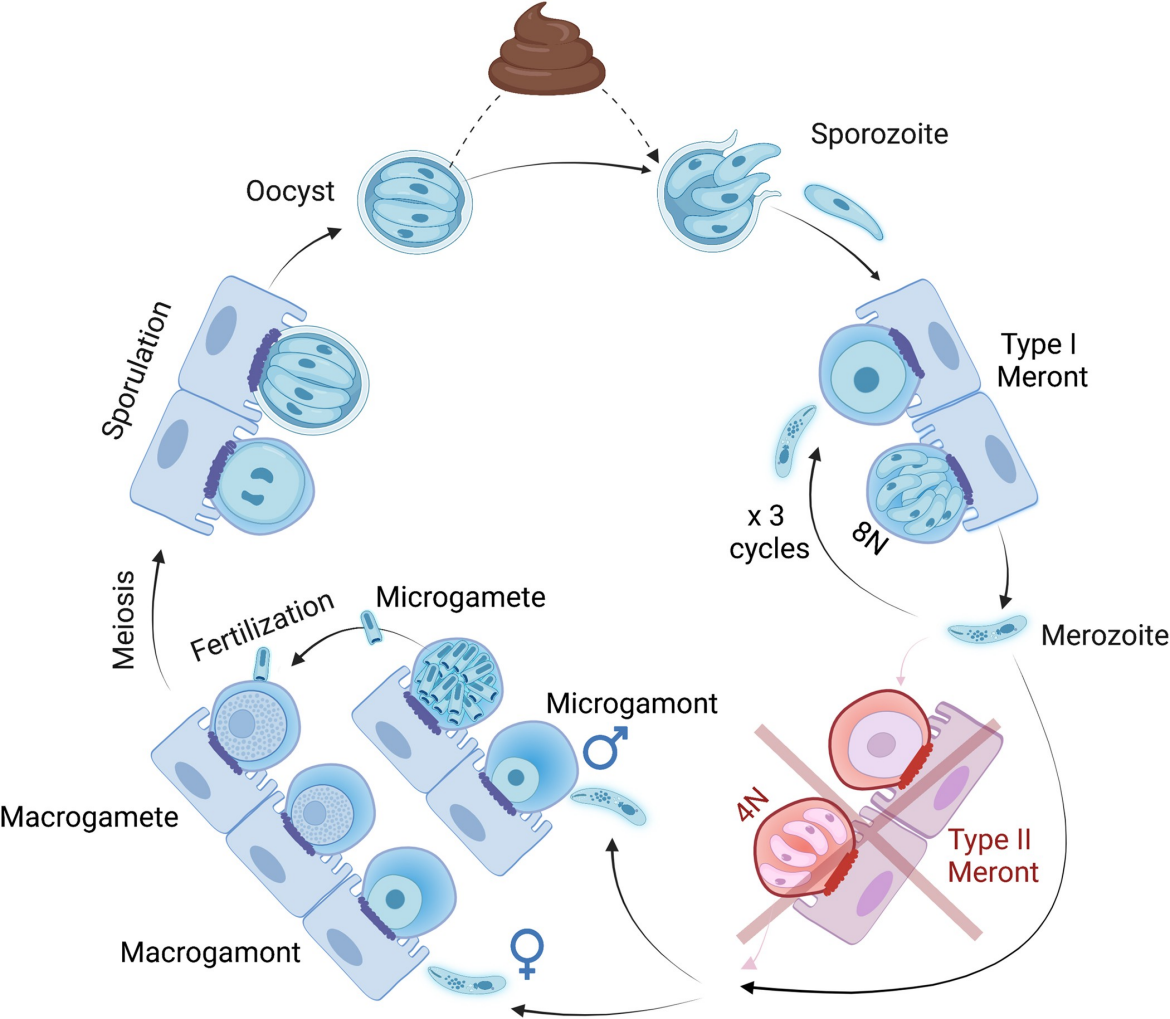
*Cryptosporidium* infection initiates by ingestion of environmentally robust oocysts, which contain 4 haploid sporozoites. Sporozoites excyst in the intestine and infect enterocytes in the intestinal epithelium. Here, parasites establish and are enclosed within a parasitophorous vacuole membrane and are separated from remainder of the host cell by an electron dense boundary. Division of this form (hitherto known as a **type I meront**) generates 8 **merozoites** (perhaps 6 in some other *Cryptosporidium* species), which egress and reinfect adjacent enterocytes where they each create 8 more merozoites. At the third such cycle, parasites differentiate into sexually committed merozoites that reinfect a new enterocyte to form a male or female **gamont**. A second proliferative cycle, involving a **type II meront** that produces 4 merozoites, was previously believed to be required for the generation of sexually committed parasites, but English and colleagues [4] find that no such form exists in their system in *C*. *parvum*. Male **microgamonts** undergo repeated mitotic divisions to each produce 16 **microgametes**, and subsequent **fertilization** between a microgamete and a female **macrogamete** results in a diploid zygote that undergoes **meiosis** to create 4 new haploid sporozoites. These sporozoites are encapsulated in an oocyst that can either be shed into the environment or can excyst to autoinfect the same host. Both thick- and thin-walled oocysts have been described microscopically, and some research suggests that only thick-walled oocysts are environmentally persistent.

Despite its global impact, there remain major gaps in our knowledge of *Cryptosporidium* biology. This is well illustrated in this edition of *PLOS Biology* in which English and colleagues [4] discover that the current understanding of the life cycle of *Cryptosporidium* is likely inaccurate (Fig 1). These discoveries were made possible by recent developments allowing the genetic modification of *Cryptosporidium* to fluorescently tag parasites, together with transcriptomic analyses to identify stage-specific genes [5,6] and live-cell imaging to track development.

Previous studies have used cell culture systems to study development of *Cryptosporidium* through its life cycle. This has involved using host cell lines, typically the colorectal adenocarcinoma HCT-8 cells. Using this model, parasites proliferate asexually for 48 to 72 hours before irreversibly transitioning to sexual gamonts [6]. Several questions arise from this, including (1) why do parasites irreversibly enter their sexual phase, at least in this model? (2) why does development stop at this point in many in vitro systems? and (3) what regulates transitions from asexual to sexual replication in *Cryptosporidium*? Answering these questions will not only help understand the basic biology of this parasite, but may also overcome obstacles to develop a much-needed continuous tissue culture system.

Using an elegant set of experiments, English and colleagues [4] start to address some of these important questions, asking first whether extrinsic or intrinsic factors drive *C*. *parvum* to undertake sexual development. They evaluate this by showing no difference in the timing of the transition to sexual development in media used for prior parasite culture compared to fresh media controls. Subsequently, using live-cell imaging with recently developed transgenic stage and sex-specific markers [6], they show that asexual and sexual cycles of *C*. *parvum* are tightly synchronized. Intriguingly, a transition to sexual commitment and development occurs, almost without exception, after 3 asexual cycles [4].

Finally, using time-lapse imaging, the authors track development of approximately 1,000 individual parasites and show that male and female gamonts (sexual stages) develop directly from 8 nuclei (8N) type I meronts (multinucleate forms that give rise to extracellular merozoites). Interestingly, each type I meront produces both male and female gamonts (slightly more females than males), indicating that sexual differentiation may be determined after type I meront formation in *C*. *parvum*. These experiments question the existence and need for previously postulated 4N type II meronts for gamont formation in *C*. *parvum*, despite the inclusion of this stage in the widely accepted life cycle for this parasite (Fig 1). English and colleagues [4] follow up on this question through additional labeling and imaging experiments of 8N and 4N cells. In their experiments, 4N cells largely progressed to 8N type I meronts, leading to merozoites that either continued the asexual phase or developed into gamonts. Although some 4N cells did not form 8N meronts, these were rare and present at stable levels throughout the experiment and expressed none of a series of marker proteins associated with merozoite or gamont formation. The authors do not conclude a role for these 4N cells. They may be the cells that others have, seemingly incorrectly, classified as type II meronts or have an as yet unknown function.

This study generates many intriguing questions. For example, if the highly synchronized and tightly timed asexual cycles are consistent with in vivo development, and there are good arguments for this presented by English and colleagues [4], how is this achieved and maintained seemingly without any extrinsic signaling? Notably, although many apicomplexans are in part driven to sexual differentiation through environmental sensing, at least one species, *Hammondia hammondi*, appears to differentiate into encysted forms through an intrinsically driven program [7]. Furthermore, what happens after 3 cycles of asexual replication to prompt a highly synchronized transition to sexual development? Such a system may include a biological clock, for example, one driven by an unknown molecule that accumulates or is depleted during asexual replication until, after 3 cycles, it reaches a critical threshold that allows sexual development. In addition, what controls the sex ratio within type I gamonts? Further, if 4N cells are not type II meronts and not required for gamont formation, what are the 4N cells observed by English and colleagues [4], and what function do they have? Techniques such as single-cell or bulk RNA sequencing (RNA-seq) on the discrete generations of meronts appear ripe to start answering some of these questions.

English and colleagues [4] provide valuable contributions to the current understanding of *C*. *parvum* development, with implications for other apicomplexans. Further, overcoming the intrinsic mechanism triggering differentiation of merozoites to sexual forms may be the key to continuous in vitro culture. As found for other apicomplexans, this differentiation could involve epigenetic factors [8] as well as AP2 [9] or myb-like transcription factors [10]. The study also strongly indicates that a significant layer of regulation, possibly involving a clock-like mechanism, governs the timing and number of asexual replicative phases of *C*. *parvum* and its transition to sexual development. Understanding the molecular biology of these phases in *C*. *parvum* has relevance for developing improved control methods. Significant research on sexual development and gametocyte formation in, for example, *Plasmodium falciparum*, a causative agent of malaria, is an important aspect of planned strategies to block parasite transmission [11]. This stage is essential for the parasite’s survival and a key target to reduce transmission. Comparatively, little is known about the mechanisms underpinning these phases in *Cryptosporidium*.
